# PLA Biocomposites Reinforced with Cinnamon-Treated Flax Fibers

**DOI:** 10.3390/ma19081632

**Published:** 2026-04-18

**Authors:** Magdalena Stepczyńska, Alona Pawłowska, Rafał Malinowski

**Affiliations:** 1 Department of Materials Engineering, Kazimierz Wielki University, JK Chodkiewicza 30 St., 85-064 Bydgoszcz, Poland; 2 Łukasiewicz Research Network—Institute of Polymer Materials, M.Skłodowska-Curie 55 St., 87-100 Torun, Poland

**Keywords:** PLA biocomposites, lignocellulosic fibers, flax fibers, trans-cinnamic acid, surface modification, interfacial adhesion, antibacterial activity

## Abstract

**Highlights:**

- A novel bio-based surface functionalization of lignocellulosic fibers using trans-cinnamic acid is proposed.
- The modification mechanism involves esterification and enhanced interfacial in-teractions with the polymer matrix
- Treatment significantly reduces fiber polarity and improves fiber–matrix adhesion.
- Functionalization leads to improved thermal behavior and structural stability of the fibers.
- The results demonstrate the potential of phenolic acid functionalization in sustainable composite design.

**Abstract:**

In this research, PLA biocomposites reinforced with 20 wt% flax fibers modified with 1, 5, 10, and 20% concentrations of trans-cinnamic acid (TC) were prepared. The materials were systematically characterized to evaluate their structural, thermal, viscoelastic, surface, and functional properties. Thermal stability and phase transitions were analyzed using thermogravimetric analysis (TG) and differential scanning calorimetry (DSC), while viscoelastic behavior and molecular relaxation processes were investigated by dynamic mechanical analysis (DMA). To elucidate failure mechanisms and interfacial quality, fracture surface morphology after tensile testing was observed using scanning electron microscopy (SEM). Surface wettability was determined through water contact angle measurements, and antibacterial activity against *Escherichia coli* and *Staphylococcus aureus* was evaluated to assess the functional potential of the developed biocomposites. The results demonstrated that moderate fiber modification improved interfacial adhesion and enhanced thermo-mechanical performance. The highest contact angles were observed for 5% and 10% TC concentrations, indicating increased surface hydrophobicity, while strong antibacterial activity (R ≥ 6) was achieved for 10% and 20% TC. The research confirms that trans-cinnamic acid concentration governs multiple structure–property relationships, enabling controlled tuning of mechanical reinforcement and antibacterial functionality.

## 1. Introduction

The growing demand for sustainable materials has intensified research into biopolymers and natural fiber-reinforced composites as environmentally friendly alternatives to conventional petroleum-based plastics. Among biodegradable thermoplastics, polylactide (PLA) has attracted considerable interest due to its renewable origin, compostability, and favorable processing properties [1]. However, PLA’s brittleness, limited thermal resistance, and moderate barrier properties limit its wider application, particularly in areas requiring improved mechanical performance and functional surface properties [2,3,4,5,6,7,8].

Many researchers focus on the use of natural-reinforcing fillers to produce completely biodegradable biocomposites. The most widely applied natural fillers are plant fibers such as hemp, flax, kenaf, bamboo, coir, sheep wool, and also other natural-based fillers such as pea pods, oak bark [9,10,11,12,13,14].

Reinforcing PLA with natural fibers such as flax fibers is an effective strategy for improving stiffness and reducing material costs while maintaining biodegradability. Nevertheless, the inherently hydrophilic nature of lignocellulosic fibers and their poor compatibility with the relatively hydrophobic PLA matrix often lead to poor interfacial adhesion, inefficient stress transfer, and premature wear. These interfacial constraints not only compromise mechanical and thermo-mechanical performance but also affect the surface properties, thermal stability, and long-term durability of the resulting biocomposites [15,16,17,18,19,20].

Surface modification of natural fibers has therefore become a key approach for tailoring fiber–matrix interactions and engineering the interphase region in PLA-based composites [21,22]. In recent years, increasing emphasis has been placed on the use of biobased and non-toxic modifiers to preserve the environmental benefits of natural fiber composites [17,23]. Trans-cinnamic acid, a naturally occurring phenolic compound derived from cinnamon, is of particular interest due to its aromatic structure, antimicrobial activity, and potential to modify surface energy and interfacial chemistry [24,25,26,27,28,29,30]. Its incorporation into fiber surface treatments offers a promising method for simultaneously enhancing interfacial adhesion, altering surface wettability, and introducing functional properties without the need for synthetic coupling agents.

Despite the growing interest in surface biomodifiers, systematic studies on the effect of modifier concentration on the thermal, thermo-mechanical, structural, and functional properties of PLA/flax fiber composites remain limited. Previous works, including our earlier studies [17,23,31,32], have primarily focused on selected aspects such as processing, mechanical performance, or surface modification efficiency [15,33,34,35,36]. However, a comprehensive understanding of how the concentration of a bio-based modifier governs interfacial interactions and multiple structure–property relationships in PLA-based biocomposites is still lacking. In particular, the balance between interfacial reinforcement and interfacial plasticization at higher modification levels is not yet fully understood. Furthermore, the combined effects of fiber modification on molecular mobility, fracture mechanisms, surface wettability, and antibacterial properties have rarely been assessed within a single, comprehensive study.

Therefore, the novelty of the present study lies in a comprehensive and integrated analysis of PLA/flax fiber biocomposites modified with varying concentrations of trans-cinnamic acid. By correlating thermal, thermo-mechanical, morphological, surface (wettability), and antibacterial properties, this work provides new insights into how modifier concentration controls competing mechanisms within the interphase region.

Importantly, this study demonstrates that trans-cinnamic acid concentration governs multiple competing interfacial mechanisms, resulting in a nonlinear relationship between interfacial adhesion, molecular mobility, thermal stability, and antibacterial performance in PLA-based biocomposites.

## 2. Materials and Methods

### 2.1. Materials

Matrix—PLA type 2003D (Cargill Dow LLC, Minnetonka, MN, USA) with a density (ρ) of 1.24 g/cm^3^, and a melt flow rate of 4.2 g/10 min (2.16 kg, 190 °C) was purchased. Biocomposites contained 80 wt% of PLA.

Reinforcement—5 mm flax fibers (Ecotex, Namysłów, Poland) were purchased. Materials contained 20 wt% of fibers.

Natural modifier—trans-cinnamic acid (TC), number CAS 140-10-3 (Sigma-Aldrich, Poznań, Poland) with a molecular weight of 148.16 g/mol. The modifier content was 1, 5, 10, and 20 wt% of TC relative to the mass of flax fibers. Considering that the composites contained 20 wt% fibers, this corresponds to approximately 0.2, 1.0, 2.0, and 4.0 wt% TC in the final PLA/flax composites.

*Escherichia coli* (ATCC 8739) and *Staphylococcus aureus* (6538 P, ATCC, Manassas, VA, USA).

### 2.2. Processing Methods

**Figure 1:**
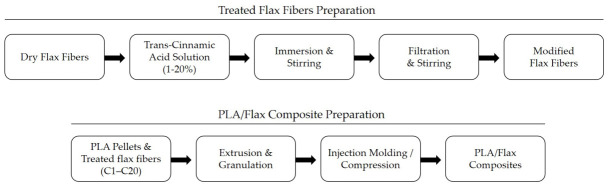
Flowchart of the fiber and composite processing process.

Figure 1 shows a block diagram of the process of obtaining the treated fibers, as well as the process of obtaining the tested composites. The processing of materials commenced with the preliminary drying of the components using a SUP-100 G laboratory dryer (WAMED, Warszawa, Poland). Flax fibers were dried at 60 °C, while PLA was dried at 50 °C. Aqueous trans-cinnamic acid (TC) solutions at concentrations of 1%, 5%, 10%, and 20% were prepared. After drying, the fibers were immersed in the prepared trans-cinnamic acid solution to induce surface modification. It should be noted that no direct spectroscopic analysis (e.g., FTIR) was performed to confirm the chemical attachment of trans-cinnamic acid to the fiber surface; therefore, the modification is interpreted in terms of adsorption and interfacial interactions. A comprehensive description of the modification procedure is provided in the Patent application No. 244804 [37] and in Ref. [17].

Subsequently, the dried components of the biocomposites (PLA, unmodified flax fibers, and TC-modified flax fibers) were accurately weighed using a laboratory scale (WPS 2100/C/2, Radwag, Radom, Poland).

The biocomposites were processed via extrusion and granulation using a co-rotating twin-screw extruder (BTSK 20/40D, Bühler, Stuttgart, Germany). Specially designed extruder screws were employed to minimize fiber damage during processing. The extrusion was carried out with cylinder heating zones set at 180 °C, 182 °C, 184 °C, and 186 °C, and a head temperature of 185 °C. The screw rotated at 120 rpm, with feed rates of 133.3 g/5 min for PLA and 33.3 g/5 min for flax fibers. The resulting biocomposites contained 80 wt% PLA (1200 g) and 20 wt% flax fibers (300 g).

After extrusion, the biocomposite granules were molded into dumbbell-shaped specimens using an injection molding machine (TRX 80 ECO 60, Tederic Machinery Manufacture, Taizhou, China) for mechanical and wettability testing. The cylinder zones of the injection molding machine were set to 170 °C, 165 °C, and 165 °C, with a head temperature of 165 °C and a mold temperature of 35 °C. The injection pressure was maintained at 24.8 MPa, resulting in specimens with a mass of approximately 11 g each.

The samples were designated as P (for neat PLA), N (PLA containing non-modified flax fibers), C1 (for sample containing fibers modified with 1% of TC), C5 (for sample contained fibers modified with 5% of TC), C10 (for sample contained fibers modified with 10% of TC), and C20 (for sample contained fibers modified with 20% of TC) (Table 1).

### 2.3. Characterization Methods

The thermal stability of the samples was assessed using a Q500 thermogravimetric analyzer (TG Q500, TA Instruments, New Castle, DE, USA). Measurements were performed over a temperature range of 25 °C to 1000 °C with a heating rate of 10 °C/min under a nitrogen atmosphere. Sample weights were between 19 and 20 mg.

Phase transition temperatures were evaluated using a differential scanning calorimeter (DSC Q200, TA Instruments, USA). The measurements were conducted from −80 °C to 200 °C at a heating rate of 10 °C/min under nitrogen. Each analysis included three stages: an initial heating, cooling, and a second heating. Only data from the second heating, after erasing the initial thermal history during the first scan and controlled cooling, were used for evaluation. Sample masses ranged from 7.2 to 8.5 mg.

The degree of crystallinity (X_c_) was calculated using the following formula:

(1) $$X_{c}=\frac{\left(\Delta H_{m}-\Delta H_{CC}\right)}{\Delta H_{m100\%}}\cdot 100\%$$

where:

ΔH_m_—the melting enthalpy, ΔH_cc_—cold crystallization enthalpy, ΔH_m100%_—melting enthalpy calculated for the pure PLA with maximum X_c_ (for pure 100% crystalline PLA ΔH_m100%_ = 93.6 J/g [38]). X_c_ was calculated based on total composite mass, including PLA, flax fibers, and TC.

Thermo-mechanical properties were investigated using a Q800 dynamic mechanical analyzer (DMA, TA Instruments, New Castle, DE, USA). Cuboid specimens with dimensions of 60 × 10 × 4 mm, cut from compression-molded samples, were tested in dual cantilever mode at a fixed frequency of 1 Hz and a controlled amplitude of 15 µm. Measurements were carried out over a temperature range of 25 °C to 160 °C.

Surface morphology was examined using a Phenom XL scanning electron microscope (ThermoFisher Scientific, Eindhoven, The Netherlands). Before imaging, samples were coated with a thin layer of gold.

The evaluation of biocidal properties was performed according to the ISO 22196:2012 standard [39]. This method assesses antimicrobial activity against two reference bacterial strains, *Escherichia coli* and *Staphylococcus aureus*. The bacterial cultures were prepared in nutrient broth and incubated for 24 h at 37 °C prior to testing. The antibacterial activity was then evaluated according to ISO 22196, under standard conditions of 37 °C and relative humidity above 90% for 24 h. The concentration of cells in the suspensions was determined using optical density measurements with a densitometer (Densitometer II, Pliva-Lachema, Brno, Czech Republic) calibrated against the McFarland scale [2]. The density of the working suspension was adjusted to 0.5 on the McFarland scale, corresponding to 1.5 × 10^8^ cells/mL.

For testing, the samples were covered with the bacterial suspensions and left for the designated periods: immediately after inoculation (0 h, for recovery efficiency validation) and 24 h. After incubation, cells were recovered from the material surfaces by suspending them in a neutralizing solution (soybean casein digest broth with lecithin). The number of viable cells was determined by inoculating Plate Count Agar (PCA). Each sample was tested in triplicate.

The antibacterial activity (R) was calculated as the logarithmic difference in viable cell counts between the biocide-containing materials and the reference material using the following formula:

R = (U_t_ − U_0_) − (W − U_0_) (2)

where U_t_ is the average common logarithm of viable cells recovered from the reference sample after 24 h, U_0_ is the average logarithm of cells recovered immediately after inoculation, and W is the average logarithm of viable cells recovered from the test samples after 24 h.

Contact angle measurements were performed using the dynamic liquid flow method with a DSA 100 goniometer (Krűss GmbH, Hamburg, Germany). In this method, a polar liquid (distilled water) was used. A drop of water was applied to the sample, and the instrument measured the dynamic contact angle of the analyzed material. For each sample, six contact angle measurements were conducted, where one measurement consisted of 96 angle readings. The number of these readings results from the volume range (V) of the liquid drop and the chosen time interval (τ) between individual contact angle measurements. The volume of liquid drops for water ranged from 3 to 7 μL. The drop flow rate and the time interval between contact angle measurements were 5 μL/min and 0.5 s, respectively.

### 2.4. Statistical Analysis

Statistical analysis of the wettability tests was conducted using a one-way analysis of variance (ANOVA) and Tukey’s post hoc test to assess the statistical significance (α = 0.05) of the effects of fiber and TC content.

## 3. Results and Discussion

### 3.1. FTIR Characterization

**Figure 2:**
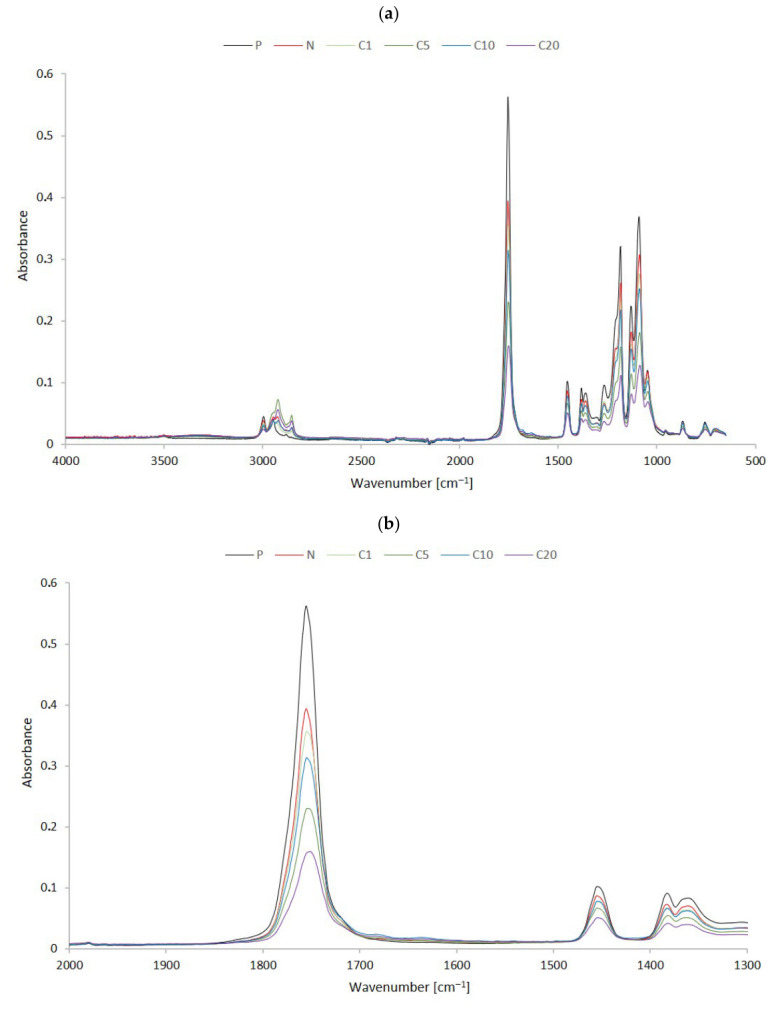

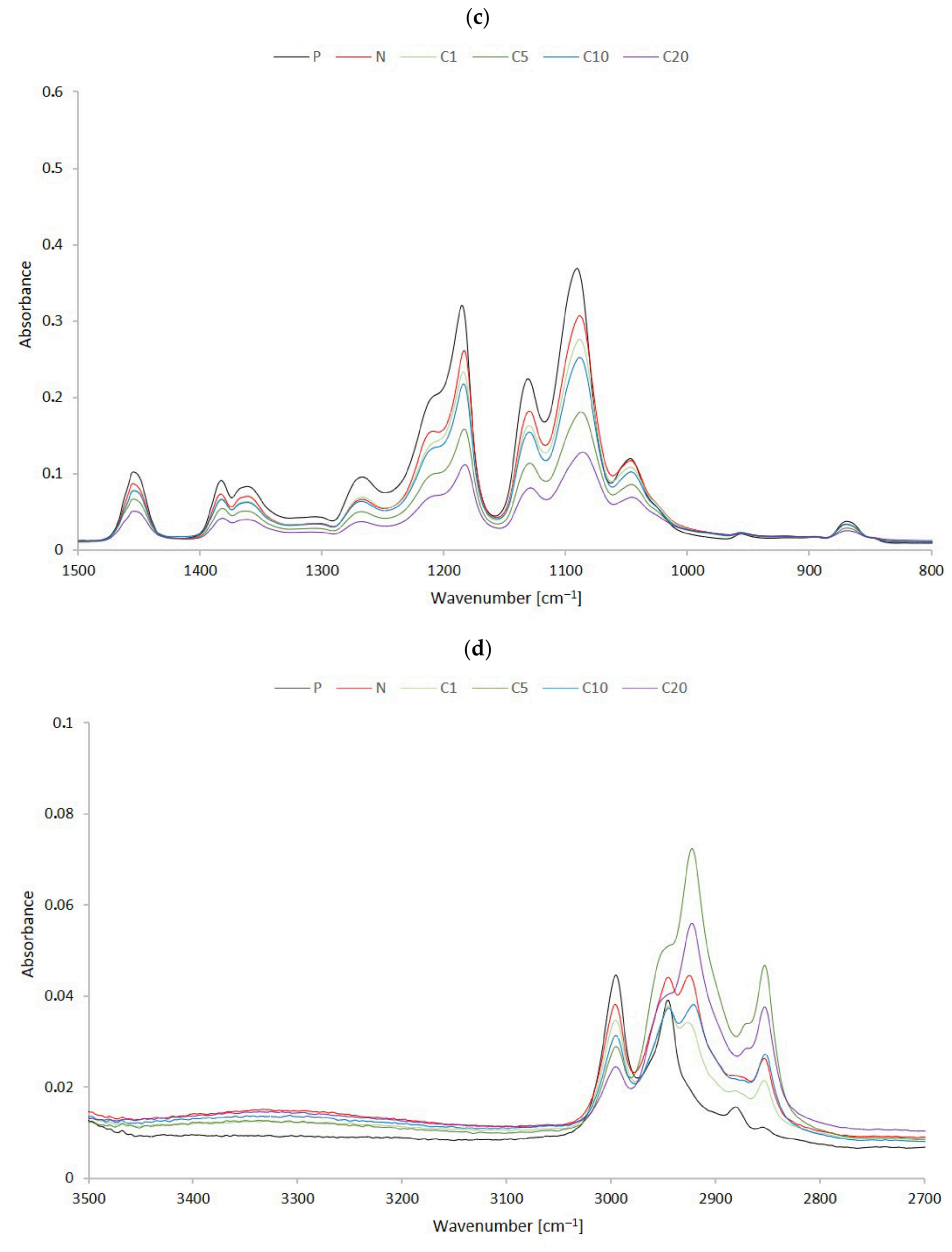
FTIR spectra of samples with selected regions (**a**) 4000–500 cm^−1^, (**b**) 1750 cm^−1^, (**c**) 1500-800 cm^−1^, and (**d**) 3500-2700 cm^−1^ Black line—P sample, red line—N sample, green line—C1 sample, olive line—C5 sample, blue line—C10 sample, purple line—C20 sample.

The FTIR spectra of neat PLA (P) and its composites with unmodified (N) and TC-treated flax fibers (C1–C20) are presented in Figure 2. All spectra show characteristic PLA absorption bands. The prominent peak at approximately 1750 cm^−1^ (Figure 2b) is attributed to the stretching vibration of the ester carbonyl group (C=O). Additional bands in the range of 1180–1080 cm^−1^ (Figure 2c) correspond to C–O–C stretching vibrations, while peaks at 2995–2945 cm^−1^ (Figure 2d) are associated with –CH_3_ stretching vibrations [2,40].

The introduction of flax fibers (N sample) causes the appearance of subtle spectral features related to lignocellulosic components, including a broad band in the range of 3300–3400 cm^−1^ (Figure 2d) attributed to O–H stretching vibrations. Small contributions in the range of 1600–1510 cm^−1^ may also be associated with aromatic structures derived from lignin [41,42].

In the case of TC-treated composites (C1–C20), no distinct new absorption bands are observed compared to the reference materials. However, there are noticeable changes in the intensity of certain bands, particularly in the region corresponding to the ester carbonyl group (~1750 cm^−1^), where a progressive decrease in peak intensity can be seen as the modifier content increases. This effect is also accompanied by variations in the regions related to –CH stretching (3000–2800 cm^−1^) and C–O–C vibrations (1180–1080 cm^−1^). These changes are primarily attributed to alterations in the relative contributions of the PLA and fiber phases, as well as modifications in the interfacial region, rather than the formation of new covalent bonds [20,21,43].

In particular, the C20 sample shows a general decrease in absorbance intensity, which may be associated with increased heterogeneity or changes in surface composition at higher modifier content.

The FTIR results indicate that treatment with trans-cinnamic acid does not create significant covalent bonds at the bulk level. Instead, the spectral changes are consistent with physical interactions, such as hydrogen bonding and surface adsorption of the modifier. This suggests that the modification mainly affects interfacial interactions rather than changing the bulk chemical structure of the composites. Further support for this interpretation comes from the DMA and SEM analyses discussed in the following sections.

### 3.2. Thermogravimetry

**Figure 3:**
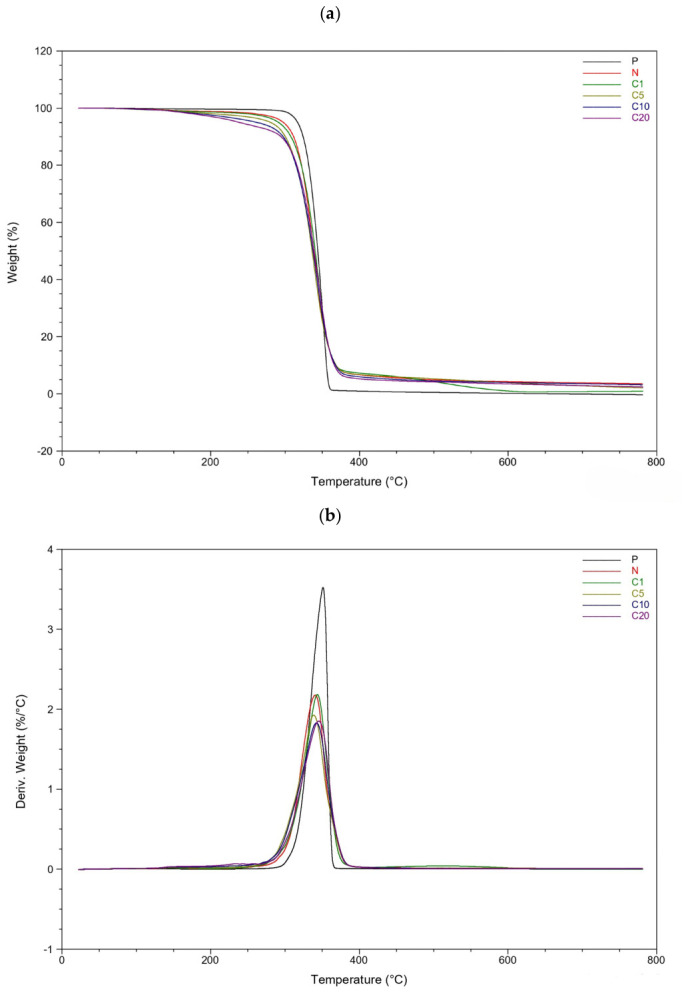
(**a**) TG curves and (**b**) DTG curves of PLA and its composites.

The results of the thermal degradation measurements for the tested samples are presented in Figure 3 and Table 2.

Sample P exhibits a single-step decomposition process, which is characteristic of neat PLA. This process involves rapid depolymerization linked to the breaking of ester bonds and the unzipping of lactic acid chains.

However, the addition of flax fibers significantly affects the onset and progression of degradation. Sample P shows a sharp mass loss occurring between 320 and 370 °C, whereas the composites degrade over a wider temperature range. This variation is due to the multicomponent nature of flax fibers, which consist of hemicellulose, cellulose, and lignin. Each of these components decomposes within different temperature intervals: hemicellulose degrades around 220 to 315 °C, cellulose between 315 and 400 °C, and lignin over a broad range that can extend up to 600 °C [15,42,44,45]. As a result, the degradation stages overlap, leading to extended mass loss profiles.

The addition of 20 wt% flax fibers (N) leads to a significant decrease in the thermal stability of the matrix. The onset degradation temperature (T5%) drops from 316.8 °C for P to 297.7 °C for N. Similarly, the temperature at which the maximum degradation rate occurs (T_d/dt_) decreases from 351.3 °C to 340.6 °C. This trend is commonly observed in PLA and natural fiber composites and is attributed to the lower thermal stability of lignocellulosic fibers, which can promote earlier thermal scission of the polymer chains. Additionally, the residual mass at 780 °C increases from 0% for P to 3.5% for N. This increase is typical of biomass-derived materials that experience partial aromatization and char formation during pyrolysis. These findings are consistent with previous reports highlighting that lignin-rich components enhance char stability at high temperatures [23].

The chemical modification of flax fibers with TC significantly impacts the degradation behavior of PLA composites. The most pronounced effect is observed in the onset temperature (T5%), which systematically decreases as the level of modification increases from 291.3 °C for C1 down to 238.6 °C for C20. This clearly indicates that the presence of TC promotes earlier thermal degradation of the composite.

Several factors may contribute to this behavior. Firstly, firstly, the chemical structure of TC includes thermally labile functionalities. Trans-cinnamic acid contains aromatic and carboxyl groups that may undergo decomposition at relatively low temperatures, initiating degradation at the fiber–matrix interface [46,47]. Secondly, catalytic interactions. The acidic groups may catalyze interactions with PLA ester bonds. Carboxylic acids can promote ester hydrolysis and transesterification when heated, which lowers the energy barriers for PLA degradation. Similar effects have been noted in PLA composites containing acidic fillers or unneutralized surface modifiers [48,49,50,51,52]. And next, enhanced interfacial adhesion. Improved compatibility between PLA and the modified fibers increases the effective contact area, potentially facilitating faster energy transfer to the polymer phase and prompting an earlier onset of chain scission.

Although T5% is significantly reduced by modification, the temperature of the maximum mass loss rate (Td/dt) remains within a relatively narrow range of 338 °C to 346 °C for all composites. This suggests that the main degradation step is still dominated by the PLA matrix and is not significantly altered by fiber modification.

All composite samples show measurable char residues ranging from 0.9% to 3.5%, whereas samples P leaves no residue at all. The highest char content is found in the unmodified composite (N). Modified samples exhibit slightly lower or intermediate levels of residue, but there is no consistent trend. This variation may be attributed to competing effects: the aromatic structure of TC may promote char formation, while acid-catalyzed degradation pathways may reduce the stability of carbonaceous residues derived from lignin. Furthermore, the temperature at which 95% degradation occurs (T95%) shifts significantly to higher values (410–520 °C) for the composite materials, indicating extended degradation related to lignin-rich fiber remnants. Notably, sample C5 achieves a T95% of 518.9 °C, the highest among all modified composites, suggesting the development of thermally stable char structures at intermediate levels of modification.

These results indicate that trans-cinnamic acid does not improve the initial thermal stability of PLA composites, but rather modifies the degradation pathway by promoting earlier onset while maintaining the main degradation process and influencing char formation at higher temperatures.

### 3.3. Differential Scanning Calorimetry

**Figure 4:**
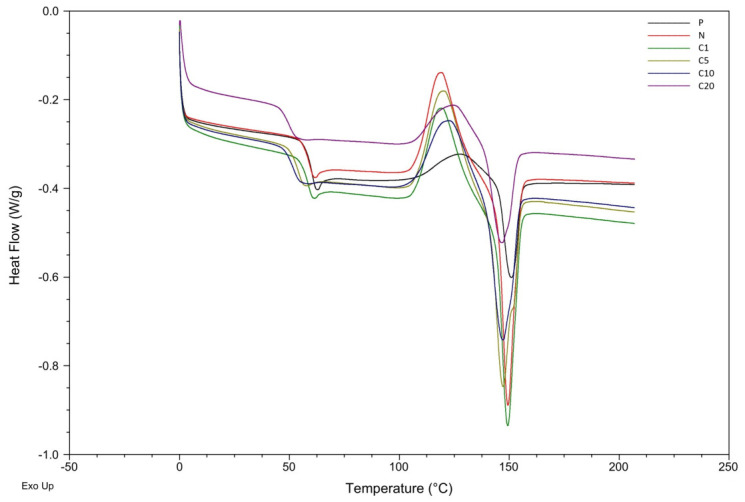
The temperature dependence on the heat flow (DSC solid curve—second heating cycle).

Figure 4 shows the DSC curves, and the obtained T_g_, cold crystallization enthalpy (ΔH_cc_), cold crystallization temperature (T_cc_), melting enthalpy (ΔH_m_), melting temperature (T_m_), and calculated degree of crystallinity (X_c_) are summarized in Table 3.

Sample P (neat PLA) shows a T_g_ of 60.4 °C, along with a moderate cold-crystallization peak at 128.1 °C and a melting peak near 151 °C. The addition of unmodified flax fibers (sample N) slightly depresses T_g_ (59.6 °C) and substantially increases both ΔH_cc_ and ΔH_m_, resulting in a crystallinity of approximately 1.9%. This indicates that lignocellulosic fibers act as effective heterogeneous nucleating agents, promoting more extensive crystallization of PLA during heating.

The samples containing trans-cinnamic acid modified fibers show progressively lower glass transition temperatures as the modifier content increases, ranging from 58.4 °C for C1 to 49.7 °C for C20. This reduction in T_g_ indicates that there is increased chain mobility, likely due to reduced interfacial compatibility or steric hindrance caused by the aromatic groups. Additionally, the presence of surface-bound trans-cinnamate moieties may disrupt the hydrogen bonding between the polymer and the fiber, resulting in increased free volume in the interphase region.

The behavior of cold crystallization shows changes. At low modifier concentrations (C1 and C5), ΔH_cc_ increases compared to neat PLA, suggesting that modified fibers initially retain their nucleating ability. However, as the modifier content increases further (C10 and C20), ΔH_cc_ decreases significantly, accompanied by a shift in T_cc_ toward higher temperatures (up to 125 °C for C20). This indicates reduced nucleation efficiency due to the partial masking of cellulose surfaces and disruption of crystalline domains by bulky aromatic groups. Concurrently, the melting enthalpy decreases with increasing modifier concentration, resulting in lower final crystallinity (ranging from 0.01% to 0.82%). This trend confirms that extensive surface modification suppresses the formation of ordered crystalline regions during heating, potentially by inhibiting chain rearrangements at the fiber–matrix interface.

The limited increase in crystallinity observed in the PLA/flax biocomposites can be explained by competing structural effects. Although flax fibers may act as heterogeneous nucleating agents that promote PLA crystallization, strong interfacial interactions between the modified fibers and the polymer matrix can restrict the mobility of PLA chains in the interfacial region. As a consequence, the ability of macromolecules to reorganize into ordered crystalline structures during cooling is partially hindered. Additionally, the relatively high fiber content (20 wt%) introduces geometric constraints and reduces the effective volume available for crystal growth. Furthermore, the presence of trans-cinnamic acid on the fiber surface may also contribute to the formation of a constrained interphase region, which can suppress long-range chain rearrangement required for extensive crystal growth. These results suggest that although flax fibers may provide heterogeneous nucleation sites, the formation of a constrained interphase region around the modified fibers limits the mobility of PLA chains and counteracts extensive crystal growth, resulting in only a minor change in the overall crystallinity of the composites. Similar behavior has been reported in other PLA–lignocellulosic fiber systems, where the nucleating effect of natural fibers is partially counterbalanced by restricted polymer chain mobility in the interfacial region, resulting in only a moderate increase in crystallinity [15,53].

### 3.4. Dynamic Mechanical Analysis

**Figure 5:**
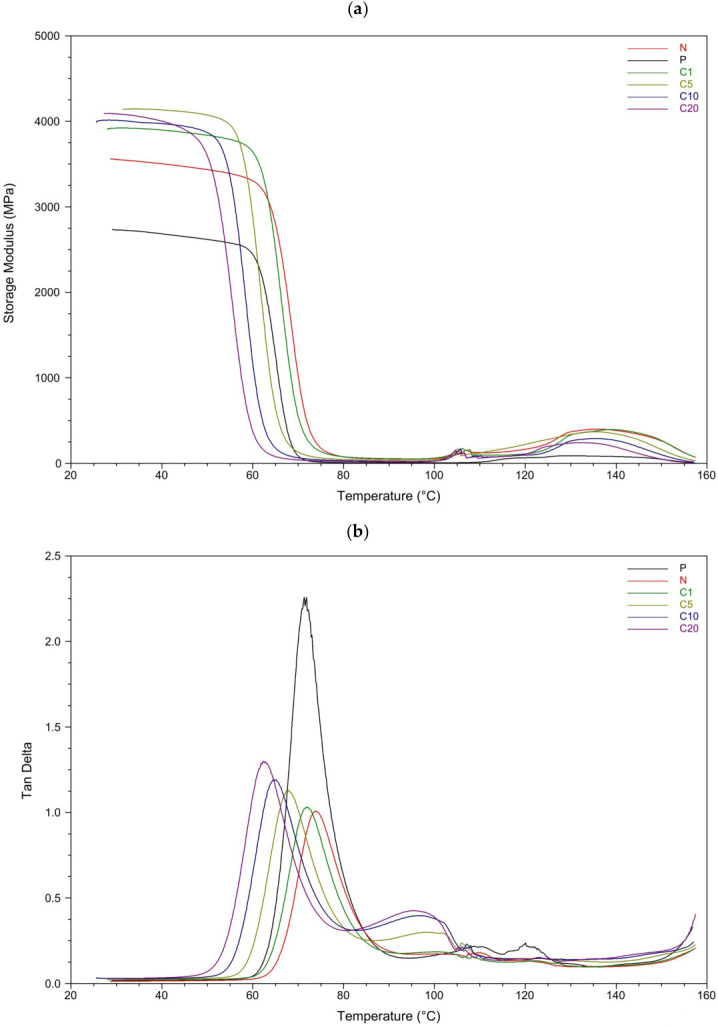
(**a**) Storage module and (**b**) damping coefficient (tan δ) of tested samples as a function of temperature.

A comparison of DMA curves shown in Figure 5 revealed the relationship between the storage modulus (E′) and the damping coefficient (tan δ) with respect to TC concentrations. Table 4 and Table 5 present thermo-mechanical parameters from DMA curves.

Although conventional tensile tests could provide complementary information on the macroscopic mechanical performance of the composites, dynamic mechanical analysis (DMA) was employed in this study to focus on the viscoelastic response and molecular mobility of the PLA matrix in the presence of modified flax fibers. DMA is widely recognized as a sensitive technique for probing viscoelastic behavior and interfacial interactions in polymer composites, since variations in the storage modulus and damping characteristics can reveal subtle changes in molecular mobility and stress-transfer mechanisms that cannot be detected by conventional static mechanical tests [54,55].

At 25 °C, the storage modulus increases from 2731 MPa for neat PLA to approximately 4100 MPa for the C5 and C20 composites, confirming a significant reinforcing effect of the flax fibers. Table 3 summarizes the storage modulus (E′) at ambient temperature. In the glassy region, neat PLA (P) demonstrates relatively high stiffness, which is typical for an amorphous polymer below its glass transition temperature; however, its storage modulus remains lower than that of all fiber-reinforced composites. Among the composites tested, C5 exhibits one of the highest storage modulus values, comparable to C20 and slightly higher than C10, while C1 shows a moderately increased stiffness. This finding confirms that moderate modification with trans-cinnamic acid leads to the most effective stress transfer between the fibers and the PLA matrix. The observed improvement is attributed to enhanced interfacial adhesion, which restricts the mobility of the polymer chains near the fiber surface and increases the composite’s effective load-bearing capacity.

In contrast, the composite with unmodified fibers (N) shows lower storage modulus values than the modified systems, indicating less efficient reinforcement due to weaker fiber–matrix adhesion and interfacial slippage. Furthermore, composites with higher levels of modification, C10 and C20, do not show a further increase in stiffness compared to C5. This trend suggests that excessive surface modification leads to the formation of a more heterogeneous and mechanically less efficient interphase, which ultimately limits reinforcement efficiency despite relatively high modulus values at low temperature. Despite the relatively high storage modulus observed for C20 in the glassy region, other results (SEM and tan δ analysis) indicate increased interphase heterogeneity at higher modifier content.

As the temperature rises toward the transition region, all materials show a pronounced decrease in storage modulus. Neat PLA undergoes a relatively sharp and well-defined transition, consistent with a homogeneous relaxation process within the polymer matrix. For composites C1 and C5, the modulus drop occurs over a slightly shifted and steeper temperature range, indicating that a significant fraction of polymer chains remains constrained to higher temperatures due to strong interfacial interactions. This behavior confirms the formation of an effective interphase that delays the onset of large-scale molecular mobility. Conversely, N shows an earlier and more gradual decrease in storage modulus, reflecting weaker fiber–matrix adhesion and earlier activation of segmental motion. In C10 and C20, the transition is broader, pointing to increased structural heterogeneity and a wider distribution of relaxation times within the over-modified interphase. In the rubbery region above the transition, composites C1 and C5 retain slightly higher storage modulus values than the other samples, indicating residual reinforcement, whereas N, C10, and C20 exhibit reduced stiffness dominated by viscous deformation.

Further insight into molecular mobility and relaxation phenomena can be gained from examining the temperature dependence of tan δ. All materials demonstrate a distinct maximum in tan δ within the temperature range typical of PLA glass transition (Table 4). This peak reflects a relaxation process linked to the cooperative segmental motion of polymer chains. The pronounced tan δ peak observed between 50 and 70 °C is attributed to the α-relaxation of PLA chains, which corresponds to the glass transition. This correlation is further supported by the close alignment between glass transition temperatures obtained from DMA and those derived from DSC, as well as by the characteristic temperature range and magnitude of the relaxation process.

Neat PLA shows the highest and sharpest tan δ peak, indicating a more pronounced viscous response and higher energy dissipation associated with cooperative chain mobility. The addition of flax fibers leads to a reduction in peak intensity and, in some cases, peak broadening, reflecting restricted molecular mobility and increased interfacial interactions. Composites C1 and C5 exhibit moderate tan δ peak heights, suggesting a balanced combination of elastic energy storage and controlled viscous dissipation, which aligns with effective interfacial bonding. In contrast, C10 and C20 display broader tan δ peaks, reflecting increased heterogeneity and the coexistence of regions with different mobility. This behavior indicates enhanced molecular friction, interfacial slippage, and a more complex relaxation environment in systems with higher modifier content.

### 3.5. Scanning Electron Microscopy

**Figure 6:**
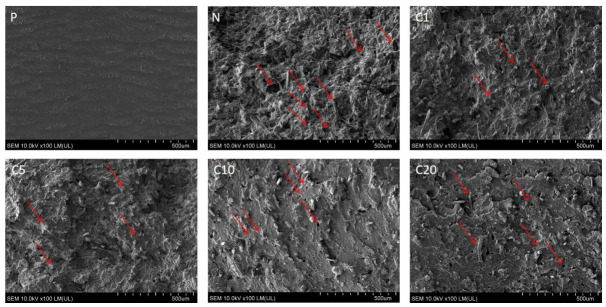
SEM micrographs. Arrows indicate characteristic features such as fiber pull-out, interfacial gaps, and regions of improved fiber–matrix adhesion.

Scanning electron microscopy (SEM) was used to examine the fracture surfaces of neat PLA (P) and PLA-based composites reinforced with unmodified (N) and trans-cinnamic acid-modified flax fibers (C1–C20) after tensile failure, as shown in Figure 6. The SEM micrographs provided valuable insights into the primary deformation and failure mechanisms, facilitating the evaluation of the fiber–matrix interfacial quality based on the degree of surface modification.

Sample P (neat PLA) shows a smooth and layered fracture surface with low roughness, typical of brittle, matrix-dominated failure. The lack of fibrillation or shear deformation suggests rapid crack propagation with minimal energy dissipation. In contrast, the composite reinforced with unmodified flax fibers (N) shows a distinctly different fracture morphology. Numerous fiber pull-outs, interfacial voids, and debonded fiber imprints are clearly visible (as highlighted by arrows in Figure 5), indicating weak interfacial adhesion between the hydrophilic flax fibers and the hydrophobic PLA matrix. The fibers appear relatively clean, with little to no matrix material adhered to their surfaces. This observation confirms that failure primarily occurred at the fiber–matrix interface, rather than through fiber fracture or cohesive matrix deformation. Such inefficient stress transfer leads to premature interfacial debonding and limits the reinforcing efficiency of the fibers, which explains the inferior mechanical performance and reduced storage modulus observed in this system.

A significant improvement in fracture morphology is observed in the composites containing fibers modified with trans-cinnamic acid at concentrations of 1% and 5% (C1 and C5). The fracture surfaces of these materials are notably rougher and more heterogeneous, with the fibers well embedded in the PLA matrix and often covered by a thin layer of adhered polymer. The lengths of fiber pull-out are reduced, and several fibers appear to be fractured instead of debonded. This suggests that the load transfer from the matrix to the fibers was sufficiently effective to cause fiber breakage. These characteristics provide compelling evidence for the formation of an effective interphase, likely resulting from enhanced chemical compatibility and intermolecular interactions due to moderate levels of surface modification.

At higher concentrations of 10 and 20% (C10 and C20), the fracture morphology becomes increasingly uneven. Although some fibers still partially bond to the matrix, significant pull-out, interfacial gaps, and microvoids were observed, especially in the case of C20. This suggests that excessive surface modification can weaken the interphase or cause plasticization, which promotes premature interfacial failure and reduces the effectiveness of reinforcement.

### 3.6. Antibacterial Activity

The antibacterial activity of the investigated materials was evaluated against two bacterial strains: *Escherichia coli* (Gram-negative) and *Staphylococcus aureus* (Gram-positive). According to ISO 22196, a material is considered biocidal if the R-value is ≥2. No biocidal activity was observed in the P and N samples. Similarly, the composite containing fibers modified with 1% TC (C1) showed no antibacterial effect (Table 6).

**Figure 7:**
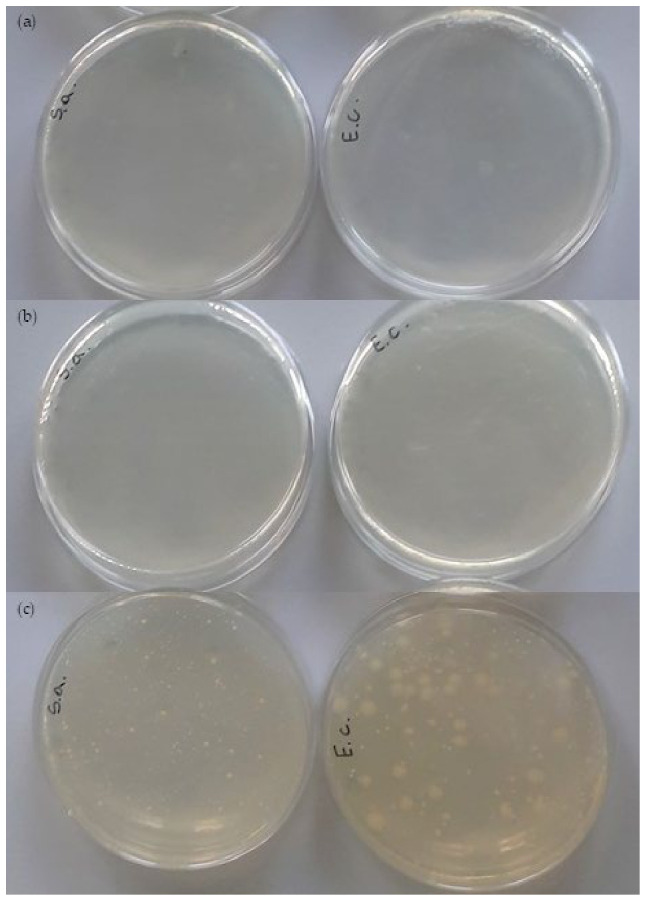
The Petri dish images: (**a**,**b**) after contact time in 10% and 20% TC (**c**) inoculation on agar (were S.a.—*Staphylococcus aureus*, E.c.—*Escherichia coli*).

A clear antibacterial response was observed in composites with higher concentrations of trans-cinnamic acid. The C5 composite demonstrated biocidal activity, achieving an R-value of 2 against both *E. coli* and *S. aureus*. In particular, the C10 and C20 composites exhibited a very strong biocidal effect, with reduction values reaching R ≥ 6 for both bacterial strains. Such high R-values indicate a strong bactericidal effect under the applied test conditions, confirming the effectiveness of the applied modification. The biocidal activity of TC is demonstrated by the images of Petri dishes containing nutrient agar (Figure 7) for both non-modified and modified samples. The C10 and C20 samples produced an inhibition zone for *E. coli* and *S. aureus* (no bacteria shown—no dots are visible).

The strong biocidal effect observed in C10 and C20 can be attributed to trans-cinnamic acid, a naturally derived phenolic compound known for its antimicrobial properties against both Gram-negative and Gram-positive bacteria [27]. The similar effectiveness against *E. coli* and *S. aureus* suggests that the antibacterial mechanism does not heavily rely on the structure of the bacterial cell wall but rather on the modifier’s ability to disrupt cellular membrane integrity and metabolic processes [28]. Increasing the concentration of the modifier likely enhances the availability of active functional groups on the composite surface, thereby intensifying the contact-mediated antibacterial effects.

Evaluating antibacterial activity is crucial for predicting which microorganisms can be inhibited and for identifying potential applications. Due to the natural origin of the modifier and the lack of toxic biocides, the composites demonstrating strong antibacterial performance (especially C10 and C20) are promising candidates for use in food-contact materials, such as active food packaging and disposable packaging applications.

### 3.7. Wettability

**Figure 8:**
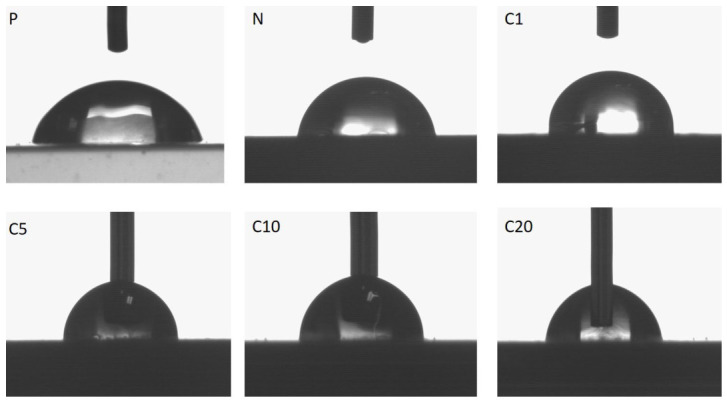
The drops of water placed on the surface of the samples.

The surface wettability of the tested composites was assessed by measuring the water contact angle (Table 7) to determine the impact of trans-cinnamic acid fiber modification. Each reported contact angle value represents the average of six droplet measurements, with each droplet value internally averaged from 96 data points collected by the goniometer software. Figure 8 illustrates the changes in the shape of a water droplet.

Neat PLA (P) showed a contact angle of 75.0°, indicating a moderately hydrophilic surface due to the presence of polar ester groups in the polymer backbone. The addition of 20 wt% unmodified flax fibers (sample N) did not significantly change the surface properties; the contact angle was 75.1°. This suggests that the PLA matrix at the surface largely obscures the hydrophilic characteristics of the fibers.

The introduction of TC-modified fibers resulted in a significant increase in the water contact angle, indicating a shift toward more hydrophobic surface behavior. The contact angle increased to 86.1° for C1, next 91.5° for C5, and achieved a maximum of 93.1° for C10. This trend is attributed to the incorporation of aromatic and nonpolar groups from trans-cinnamic acid, which reduces surface free energy and limits interactions between water and the surface. In C20, a slight decrease in the contact angle to 90.2° was observed, likely due to increased surface heterogeneity and partial exposure of polar functional groups resulting from an excessive concentration of the modifier.

It is important to note that results in surface wettability do not directly correlate with the bulk fracture morphology observed through SEM. While SEM showed relatively uniform interphase and fracture surfaces for C1 and C5, C10 demonstrated the beginning of heterogeneity, and C20 exhibited significant interphase degradation. The high contact angle for C10 indicates effective functionalization at the composite surface, even though the bulk interphase integrity starts to decline. This underscores that surface hydrophobicity is influenced by surface chemistry, while bulk fracture morphology and interfacial uniformity depend on the distribution and concentration of the modifier within the interphase. In the case of C20, excessive modification negatively impacted both surface uniformity and interphase cohesion, resulting in a moderate reduction in hydrophobicity and inferior mechanical performance.

The effectiveness of the antibacterial agent varies with concentration. Although C5 showed increased hydrophobicity, its biocidal activity only reached the threshold level (R = 2). In contrast, both C10 and C20 exhibited very strong antibacterial performance (R ≥ 6) against *Escherichia coli* and *Staphylococcus aureus*. This suggests that antibacterial effectiveness is more closely related to the total concentration and availability of active trans-cinnamic acid groups than to surface hydrophobicity alone. While greater hydrophobicity may help enhance contact between bacterial cells and the composite surface, the extent of bacterial reduction largely depends on the concentration of bioactive functional groups.

Overall, these findings show that the optimal surface hydrophobicity (C5–C10), maximum antibacterial activity (C10–C20), and optimal interfacial integrity do not necessarily occur at the same concentration of the modifier. Instead, the concentration of trans-cinnamic acid influences several structure–property relationships. This affects surface chemistry, interphase cohesion, thermo-mechanical stability, and functional antibacterial performance through mostly independent mechanisms. Therefore, future studies will focus on long-term stability, controlled release behavior of the bioactive modifier, and advanced interphase characterization to better balance mechanical reinforcement and antibacterial functionality.

Statistical analysis using one-way ANOVA followed by Tukey’s HSD test revealed statistically significant differences among all modified samples (*p* < 0.05). However, no significant difference was found between the P and N samples. The low standard deviations also support the reliability and reproducibility of the observed trends.

It is important to note that the measured water contact angle primarily reflects the composition of the outermost surface of the composite, which is largely influenced by the PLA matrix due to the processing-induced encapsulation of the fibers. However, at higher levels of modification, partial exposure of the fibers or the presence of modified interfacial regions may also contribute to the observed changes in wettability. Consequently, the contact angle values reflect the combined influence of the matrix surface properties and localized fiber-related heterogeneities.

## 4. Conclusions

PLA-based biocomposites reinforced with 20 wt% flax fibers were prepared using various concentrations of trans-cinnamic acid (1%, 5%, 10%, and 20%). These composites were then evaluated for their thermal, thermo-mechanical, morphological, surface, and antibacterial properties. The results indicate that modifying the fiber surface significantly influences the interphase structure and the overall performance of the composite.

Thermal analysis indicated that fiber modification affected the composites’ degradation behavior and crystallization. Although the onset degradation temperature decreased with increasing modifier content, the main degradation process remained largely governed by the PLA matrix. Dynamic mechanical analysis revealed that composites with moderately modified fibers, particularly C5, displayed a higher storage modulus and reduced molecular mobility. This indicates improved interfacial adhesion and more efficient stress transfer. In contrast, excessive modification (C20) resulted in interphase heterogeneity and decreased mechanical efficiency.

Scanning electron microscopy revealed that the fracture mechanisms were significantly influenced by the level of modification applied. Composites with a moderate amount of modifier exhibited enhanced fiber–matrix cohesion and less fiber pull-out. In contrast, higher concentrations of the modifier led to more interfacial defects and a more heterogeneous fracture morphology.

Antibacterial testing demonstrated that biocidal properties increased with modifier concentration. While 5% TC concentration reached the threshold of biocidal activity (R = 2), composites with 10% and 20% TC concentration exhibited strong antibacterial effectiveness (R ≥ 6) against both *Escherichia coli* and *Staphylococcus aureus*.

Water contact angle measurements showed a significant increase in surface hydrophobicity for modified composites, with maximum values observed for 5% and 10% TC concentrations. However, surface wettability did not directly correspond to bulk interphase integrity, highlighting the distinction between surface chemistry and structural reinforcement mechanisms.

The study confirms that modifying PLA biocomposites with trans-cinnamic acid enables controlled engineering of the interphase, specifically in flax fiber-reinforced composites. This approach allows for independent adjustment of mechanical reinforcement and antibacterial properties.

The results indicate that no single modifier concentration provides a universally optimal performance across all evaluated properties. Instead, the observed trends reflect a trade-off between mechanical reinforcement and antibacterial functionality.

In particular, composites containing 5% trans-cinnamic acid exhibit the most favorable balance in terms of interfacial adhesion and thermo-mechanical performance, making them suitable for structurally demanding applications. In contrast, higher modifier contents (10–20%), although associated with some reduction in mechanical efficiency, provide significantly enhanced antibacterial activity, making them more attractive for active packaging applications. Therefore, the optimal modifier concentration should be selected depending on the targeted application rather than considered as a universal optimum.

As a result, these materials show strong potential for use in sustainable and active packaging applications, where the balance between mechanical integrity and antibacterial performance can be tailored through modifier concentration.

Future research will focus on evaluating the long-term stability of the tested biocomposites. The plan is to examine the impact of aging on selected properties of biocomposites that contain TC.

## Figures and Tables

**Table 1:** The description of the sample labels.

Sample	PLA Content [%]	Flax Fibers Content [%]	TC Solution Used [%]
P	100	0	0
N	80	20	0
C1	80	20	1
C5	80	20	5
C10	80	20	10
C20	80	20	20

**Table 2:** Thermal properties by TGA of biocomposites.

Sample	T_d_ (°C)	T_d/dt_ (°C)	T_5%_ (°C)	T_50%_ (°C)	T_95%_ (°C)	Residue at 780 °C (%)
P	330.95	351.32	316.80	343.98	357.64	0
N	317.76	340.56	297.65	338.56	489.64	3.5
C1	320.75	343.79	291.29	340.46	480.64	0.9
C5	313.00	338.41	280.57	336.29	518.88	2.0
C10	314.02	342.71	261.89	337.44	451.75	3.1
C20	313.38	345.40	238.64	339.02	412.31	2.4

**Table 3:** Results obtained during DSC tests of biocomposites.

Sample	T_g_ [°C]	ΔH_cc_ [J/g]	T_cc_ [°C]	ΔH_m_ [J/g]	T_m_ [°C]	X_c_ [%]
P	60.40	7.74	128.14	8.51	150.95	0.77
N	59.55	20.02	119.14	21.91	149.41	1.89
C1	58.41	20.03	119.54	20.04	149.24	0.01
C5	54.16	22.37	120.53	22.73	147.02	0.36
C10	52.02	17.59	122.80	147.00	18.41	0.82
C20	49.73	11.05	124.62	146.62	11.45	0.4

**Table 4:** Storage modulus (E′) at 25 °C temperature.

Sample	E′ (25 °C) [MPa]
P	2731
N	3561
C1	3960
C5	4141
C10	4050
C20	4092

**Table 5:** The glass transition (T_g_) temperature and damping coefficient (tan δ) peak temperature.

Sample	T_g_ [°C]	tan δ
P	71.61	2.226
N	73.87	1.008
C1	71.89	1.029
C5	67.80	1.127
C10	64.90	1.189
C20	62.43	1.297

**Table 6:** The antibacterial activity of the investigated materials.

Sample	*Escherichia coli* [CFU/mL]		*Staphylococcus aureus* [CFU/mL]	
	T_1_	R	T_1_	R
P	1.5 × 10^7^	0	2.0 × 10^7^	0
N	1.5 × 10^7^	0	1.5 × 10^7^	0
C1	1.5 × 10^6^	0	2.0 × 10^6^	1
C5	3.0 × 10^5^	2	7.5 × 10^5^	2
C10	≤1.0 × 10^1^	≥6	4.0 × 10^1^	6
C20	≤1.0 × 10^1^	≥6	≤1.0 × 10^1^	≥6

T_0_—the numbers of cells of the tested strains (1.5 × 10^8^ jtk); T_1_—number of bacterial cells after contact time. The time of the contact of bacteria with the tested foil was 24 h.

**Table 7:** The water contact angle of the tested composites.

Sample	P	N	C1	C5	C10	C20
ϴw	75.0 ± 0.3	75.1 ± 0.4	86.1 ± 0.4	91.5 ± 0.2	93.1 ± 0.5	90.2 ± 0.3

Values represent mean ± standard deviation (n = 6).

## Data Availability

The original contributions presented in this study are included in the article. Further inquiries can be directed to the corresponding author.
